# Comparative transcriptomics of salinomycin molecular toxicity in chicken and turkey

**DOI:** 10.1038/s41598-025-08812-7

**Published:** 2025-07-01

**Authors:** İlksen Berfin Ekinci, Anna Sławińska, Kacper Żukowski, Małgorzata Olejnik

**Affiliations:** 1 Department of Basic and Preclinical Sciences, Institute of Veterinary Medicine, Faculty of Biological and Veterinary Sciences, Nicolaus Copernicus University in Toruń, Gagarina 7, 87-100 Toruń, Poland; 2 Department of Cattle Breeding, National Research Institute of Animal Production, Krakowska 1, 32-083 Balice n. Kraków, Poland

**Keywords:** Salinomycin toxicity, Comparative toxicogenomics, RNA-seq, Species susceptibility, Chicken, Turkey, Molecular biology, Transcriptomics

## Abstract

Salinomycin (Sal) is an antiparasitic agent used in veterinary medicine and is characterized by low therapeutic index and high toxicity. Among poultry, chickens are resistant to Sal toxicity, but turkeys are considered susceptible. However, underlying mechanisms of Sal toxicity are poorly understood. This comparative transcriptomic study aimed to determine molecular toxicity mechanisms of Sal in both species. We conducted two experiments on chickens and turkeys exposed to Sal (0.9 mg/kg b.w/day) vs. unexposed. Heart and liver (n = 6) were collected post-mortem (chicken 5th; turkey 13th week). RNA was isolated and examined by RNA-seq to identify differentially expressed (DE) genes and pathways. Number of significant DE genes in chicken was 673 (heart) and 3049 (liver), and in turkey, 485 (heart) and 2337 (liver). Enrichment analysis revealed that Sal exposure activated platelet signaling in chicken heart, while it induced cell cycle arrest in turkey heart. In liver, impaired Sal biotransformation was determined as a shared response. In turkey liver, we determined that extracellular matrix pathway was upregulated, which could indicate liver fibrosis. Our findings demonstrate that molecular toxicity of Sal differs between species and turkey confirmed being more susceptible to Sal toxicity also at molecular level via induced cell cycle arrest and fibrosis.

**Supplementary Information:**

The online version contains supplementary material available at 10.1038/s41598-025-08812-7.

## Introduction

Salinomycin (Sal) belongs to mono-carboxylic polyether ionophores and is responsible for cation (especially K^+^ and Na^+^) flux throughout the cellular membranes^1^. It is used to control coccidiosis, a parasitic disease in animals^2^. It has been reported that the accidental use, misuse, or interaction of Sal with other drugs generates toxicity in the host organism, and significant clinical findings are characterized by muscle weakness, diarrhea, decreased feed consumption, poor weight gain, and death^3–5^.

Heart and skeletal muscles are the target organs of Sal toxicity. Histopathology observations after Sal exposure include myocardium fiber degeneration, cardiomyopathy. Clinical signs may involve tachycardia, and heart attack^6,7^. Furthermore, induced skeletal muscle lesions are observed in Sal intoxication^8^. The increased levels of hepatic tissue damage-related biomarkers such as AST and ALT in serum indicate the injury of the liver where Sal undergoes biotransformation^9–11^. At the molecular level, the excessive Sal concentration increases reactive oxygen species (ROS) and elevates intracellular Ca^2+^ influx^1,12^. A study performed by Gao et al.^13^ has demonstrated that Sal inhibited oxidative phosphorylation and triggered mitochondria-related apoptosis in-vitro. In consequence, it caused mitochondrial injury in the primary chicken myocardial cells in a dose-dependent manner. Similarly, it has been determined that Sal reduced cell viability via apoptosis in the chicken hepatoma cell line^14^. In addition, a recent study showed that Sal-induced ROS-associated mitophagy in porcine jejunal cells^15^.

The recommended dose of Sal depends on animal species. For instance, in chickens, it is determined as 50–70 mg/kg feed^16–18^, whereas rabbits tolerate Sal doses up to 25 mg/kg feed^18,19^. Among poultry, Sal is widely used as a feed additive deemed safe for chickens. However, even low concentrations of Sal, such as 9, 16, and 24 mg/kg feed, were highly toxic in turkeys, an important food source^20–22^. In addition, the median lethal dose (LD_50_) of Sal is between 40 and 44.3 (mg/kg b.w.) in chicken^18^, whereas it is determined as low as 0.6 (mg/kg b.w.) in turkey^16^. Therefore, chickens are considered the most resistant species, whereas turkeys are the most susceptible among poultry. This species susceptibility has increased interest in developing targeted veterinary solutions, such as vaccines designed specifically for chickens and turkeys^23^.

Transcriptomics is a commonly used next-generation sequencing (NGS) approach for sensitively evaluating dynamic changes in global gene expression profiles after xenobiotic exposure in biological systems. Furthermore, it provides valuable insight into the mechanism(s) underlying the xenobiotic toxicity^24^. Using transcriptomics in-vitro, studies demonstrated molecular toxicity mechanisms of ionophores in chickens. These studies showed that different ionophores dysregulated common molecular mechanisms^13,25^. According to our best knowledge, no in-vivo comparative transcriptomics study has focused on Sal’s molecular toxicity mechanisms in turkeys and chickens.

Determining Sal’s molecular toxicity would help address extensive ionophore molecular toxicity mechanisms across poultry. Also, clarifying the Sal intoxication mechanisms in chickens and turkeys would help to identify novel therapeutic approaches to eliminate or minimize the toxicity effects and improve the animals’ welfare.

This study aimed to understand the Sal toxicity mechanism at the molecular level in chicken (Gallus gallus) and turkey (Meleagris gallopavo). For this purpose, we performed transcriptome sequencing (RNA-seq) to identify differentially expressed (DE) genes and associated pathways in chicken and turkey heart and liver tissue in control and Sal-exposed.

## Materials and methods

### Experimental design and animal housing

The experiments were conducted on chicken and turkeys according to Approval No. 25/2022 of the Local Ethics Committee of Animal Experimentation in Bydgoszcz. All experiments were reported in accordance with ARRIVE guidelines (https://arriveguidelines.org) and were carried out in accordance with relevant guidelines and regulations. One-day-old female broiler chickens and turkeys were purchased from commercial hatcheries. Birds were kept on litter in pens with an area of 3 m^2^ divided into pens. The broiler chickens were housed at a density of 10 individuals per pen, whereas turkeys were housed at 4 individuals per pen.

### Diet treatment

During the pre-experimental period (chickens weeks 1–3 and turkeys weeks 1–11), birds were fed Sal-free diets. They were monitored every day, and clinical observation was performed once a week. After the pre-experimental period, birds were selected randomly and classified into two groups: the Sal-exposed and the control group. The Sal-exposed group received an experimental diet with Sal ad libitum. Experimental feeds were prepared by Zoolab (Sedziszow, Poland). Sacox 120 was used as a source of Sal. The intermediate premixture containing 500 mg/kg Sal in ground turkey feed was mixed with complete feed and granulated. The concentration of salinomycin was verified with LC–MS-MS^26^ and HPLC-UV^27^. The feeds for rearing period and control groups did not contain salinomycin above 0.073 mg/kg. The feeds for turkeys and chickens were found to contain Sal at concentrations 21.4 mg/kg and 10.9 mg/kg, respectively. Sal was administered in-feed at the concentration of 10.5 mg/kg feed in chicken during weeks 4–5 and 21 mg/kg feed in turkey during weeks 12–13 in turkey. The estimated daily dose of Sal was 0.9 mg/kg b.w. in both species. According to the literature, this dose is of the same order of magnitude as median lethal dose (LD_50_) for turkey species^28^. The control group received Sal-free diets ad libitum. Each batch of feed was tested using an in-house developed and validated method^26^.

### Sampling

**Fig. 1:**
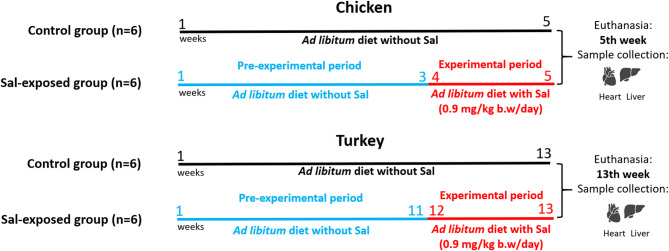
Experimental design of Sal toxicity on chicken and turkey.

On the last day of the experiment, six animals (n = 6) from the Sal-exposed and control groups of each species were euthanized by cervical dislocation after isoflurane sedation. The euthanasia method was carried out in accordance with the relevant guidelines^29,30^. The heart and liver tissues were dissected, cut into small pieces, and preserved in a fixRNA solution (EURx, Poland) before storing at − 80 °C. The experimental design is summarized in Fig. 1.

### RNA isolation

The total RNA was isolated from the liver and heart samples using the Maxwell® RSC instrument (Promega, USA). The tissue size was 100 mg, and the extraction was performed with Maxwell® RSC simplyRNA Tissue Kit (Promega, USA). Due to differences in the tissue structure, homogenization was performed in a different manner for liver and heart samples. The liver tissue was homogenized with a handheld homogenizer directly in a lysis buffer included in a kit. Because of the low cell density and fibrotic structure of the heart tissue, RNA Extracol (EURx, Gdansk, Poland) was used for homogenization, followed by the manufacturer’s protocol. The total RNA quantity and quality were estimated fluorometrically by QuantiFluor® RNA System kit (Quantus™ Fluorometer, Promega, USA). The purity of RNA was determined with the ND-2000 spectrophotometer (Thermo Fisher Scientific, USA). The integrity of rRNA bands was estimated on Agilent Bioanalyzer 2100 (Agilent Technologies, USA) using RNA Nano 6000 Lab chip kit (Agilent Technologies, USA). Samples with RNA integrity (RIN) above 7 were selected for RNA-seq. 48 samples (6 samples from each tissue per species) were chosen for RNA-seq.

### Library preparation and RNA-seq

The Hieff NGS Ultima Dual-mode mRNA Library Prep Kit (Yeasen Biotechnology, China) was used to construct libraries for 48 samples. Products were purified with Hieff NGS DNA selection Beads (Superior Ampure XP alternative) (Yeasen Biotechnology, China). The qualitative and quantitive control of libraries were determined with Qsep-400 (BiOptic Inc., Taiwan) using Qsep400 standard DNA clip (BiOptic Inc., Taiwan) and Qubit 3.0 Fluorometer (Thermo Fisher Scientific, USA) using Qubit dsDNA HS Assay Kit (Thermo Fisher Scientific, USA) respectively. The cDNA libraries were sequenced on the Illumina Novaseq 6000 platform (Illumina, USA) using the NovaSeq 6000 S4 Reagent Kit (Illumina, USA), and PE150 bp NGS data were generated.

### Quality control of the reads

The initial sequence data underwent quality assessment using FastQC^31^. Following this, the fastp software^32^ was used to remove Illumina adapters and poly-A sequences. Additionally, sequences shorter than 36 bp and those with a quality score below 20 were discarded.

### Mapping

The cleaned sequences were then mapped to the *Meleagris gallopavo* genome (Turkey_5.1.110) and the *Gallus gallus* genome (bGalGal1.mat.broiler.GRCg7b) using reference annotations that included 16,226 and 17,007 genes from the Ensembl database for turkey and chicken, respectively.

### Quantification of gene expression level

Gene-level expression was quantified using the RSEM package^33^, in combination with the STAR aligner^34^. Alignment and differentially expressed gene statistics were produced with SAMStat^35^ and RNA-SeQC^36^. For visualizing raw sequence data and compiling results for all samples into a single report, we used the MultiQC bioinformatics tool^37^.

### Differential gene expression analysis

Differentially expressed genes and their associated *p*-values were identified using DESeq2^38^. To control the false discovery rate due to multiple testing, all *p*-values were adjusted accordingly. A threshold for statistical significance was set at an adjusted *p*-value of ≤ 0.05 for identifying differentially expressed genes. Additionally, batch effects were corrected using the sva (Surrogate Variable Analysis) package^39^ to ensure robust differential expression results. Transcripts exhibiting a log fold change in gene expression of 1.0 or more were included in further analysis.

### Gene annotation and functional gene set enrichment analysis

For the gene annotation, we used GProfiler^40^ (https://biit.cs.ut.ee/gprofiler/gost), which is an online tool that determines ortholog genes between species based on information from the Ensembl database (https://www.ensembl.org). All DE genes were translated to *Homo sapiens* (human) genes using the Ensembl gene identification number. The Gene Ontology (GO) analysis was performed with EnrichR^41^. The gene set enrichment analysis was performed with the Reactome functional database using Webgestalt^42^, and *Homo sapiens* was selected as an organism of interest. Significance was set as *p* ≤ 0.05, and the normalized enrichment score (NES) was assessed.

### Reverse transcription-quantitative PCR (RT-qPCR)

Twenty-two DE genes were selected to validate the reliability of RNA-seq data using RT-qPCR. In each tissue, selected genes were involved in the most enriched pathways and belonged among the top ten differentially expressed between the Sal-exposed and control groups according to the RNA-seq data. We used the RefFinder^43^ to produce a comprehensive ranking of candidate housekeeping genes separately for each tissue/species dataset. RefFinder ranked glucose-6-phosphate dehydrogenase (*G6PDH*) (BestKeeper Index: r = 0.956, *p* < 0.001) and actin beta (*ACTB*) (r = 0.973, *p* < 0.001) highest in chicken heart and liver, phosphoglycerate kinase (*PGK1*) (r = 0.910, *p* = 0.001) and succinate dehydrogenase complex flavoprotein subunit A (*SDHA*) (r = 0.978, *p* < 0.001) highest in turkey heart and liver. This context‐specific selection ensures normalization is based on the least variable genes within each group. The housekeeping gene selection was based on their common use in the literature (Supplementary Table S1). Primer sequences were designed using Primer-Blast^44^. Primer sequences of target and housekeeping genes are given in Supplementary Table S1. 2.5 μg RNA was reverse transcribed from each sample into complementary DNA (cDNA) using the Maxima First Strand cDNA Synthesis Kit for RT-qPCR (Thermo Fisher Scientific, USA), following the manufacturer’s recommendations. Obtained cDNA was diluted to 100 ng/μL and stored at − 20 °C. The qPCR was carried out with Maxima SYBR Green qPCR Master Mix (Thermo Fisher Scientific, USA) in a 10 μL reaction volume including 2 μL diluted cDNA, 1 μM forward and 1 μM reverse primer. Each qPCR reaction was conducted in six biological replicates for chicken heart and liver Sal-exposed and control groups, turkey heart Sal-exposed and control groups, turkey liver Sal-exposed group and it was conducted in five biological replicates for turkey liver control group. Each reaction was performed in two technical replicates. Thermal cycling was performed with the LightCycler II 480 (Roche Diagnostics, Switzerland). The steps were set as initial denaturation (15 min at 95 °C), followed by 40 cycles of denaturation (15 s at 95 °C), annealing (15 s at 58 °C), and extension (30 s at 72 °C). Melting-curve analyses were conducted at the end of amplification. The mRNA levels of DE genes were normalized using the average cycle threshold (Ct) values of *ACTB-G6PDH* and *PGK1-SDHA* housekeeping genes. The relative mRNA expression values of DE genes between the Sal-exposed and the control group were calculated using the 2^−ΔΔCt^ method.

### Statistical analysis

Sample size selection was calculated and confirmed with G*Power3.1 software^45^. Given sample (n = 6) and effect size and power 0.80 (1−β err prob) showed that selected sample size was adequate for this study for each species. Relative mRNA expression of DE genes between Sal-exposed and control groups for each tissue was calculated with MS Excel. The statistical analysis of RT-qPCR data was performed with GraphPad Prism 5 (www.graphpad.com) using an unpaired *t-test*, and *p* ≤ 0.05 was considered statistically significant. The Principal Component Analysis (PCA), volcano plot, Gene Ontology (GO), enrichment analysis, and heatmap were created with SRplot^46^.

## Results

### RNA-seq data analysis

RNA-seq resulted in a minimum yield of 2.22 Gb sequencing data per sample. Samples were considered by total reads, unique alignment (%), Q30 (%), and GC content (%). Around 43 to 61 million total reads for chicken and 9 to 53 million total reads for turkey were generated. GC content was between 47–52% and 45–52% in chicken and turkey, respectively. The percentage of Q30 in each sample was above 93%. Detailed data for each sample is provided in Supplementary Table S2 for chicken and Supplementary Table S3 for turkey. The quality control of RNA-seq showed that one sample (sample code: TLV_Control_3, Table S3) from the liver control group in turkey did not provide high-quality data (9.4 million total reads and 6.1 million unique alignments) compared to others. We excluded this sample from the turkey liver control group, and further analyses were performed with 47 samples.

**Fig. 2:**
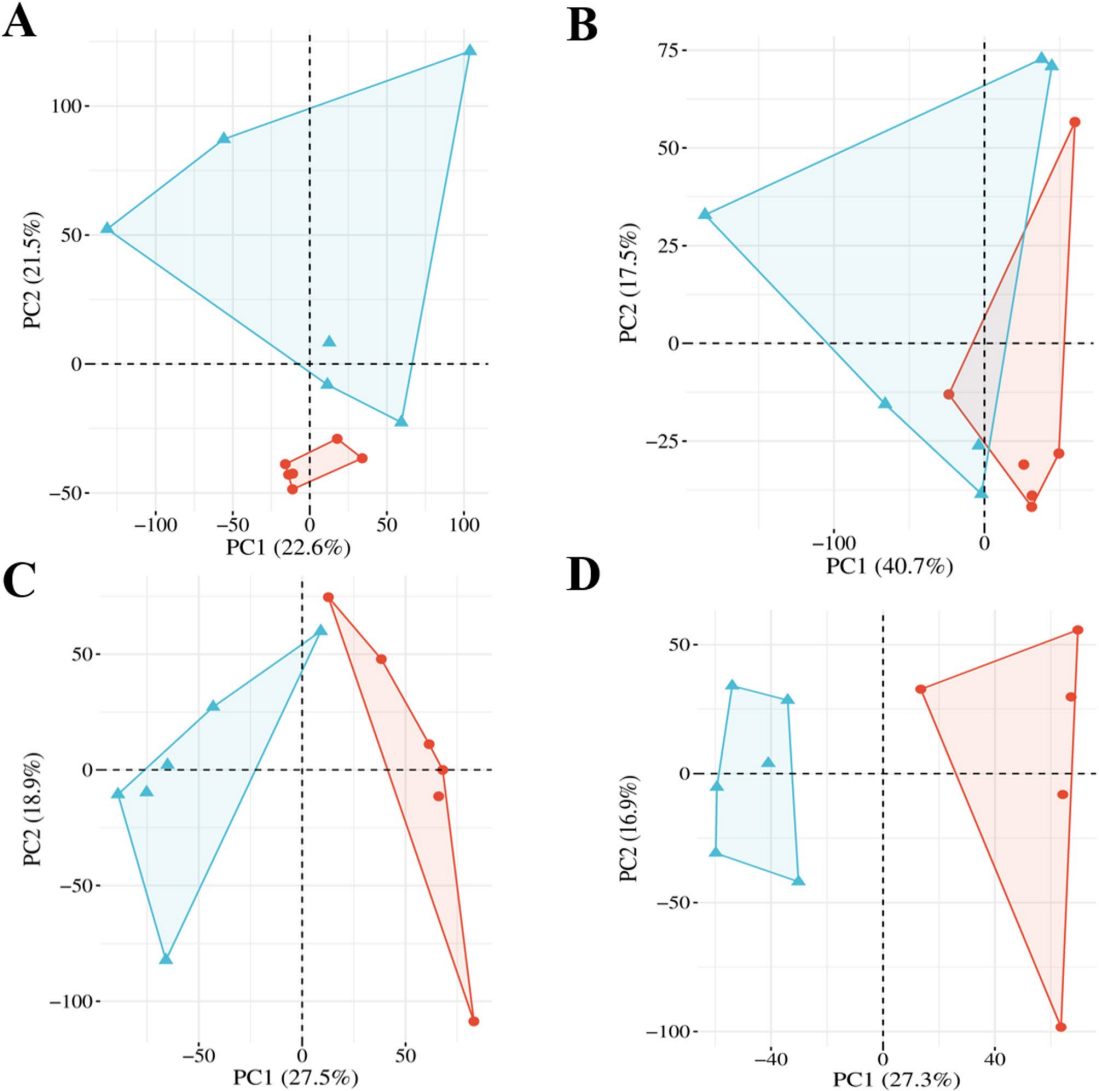
The Principal Component Analysis (PCA) in Sal-exposed and control groups in both species. PCA was performed using normalized counts of DE genes for chicken heart (**A**), turkey heart (**B**), chicken liver (**C**), and turkey liver (**D**). Each point represents an individual biological replicate. Control samples are shown as red circles; Sal-exposed samples as blue triangles. The percentages of variance explained by PC1 and PC2 are shown on the X and Y axes, respectively.

The PCA was performed to investigate whether samples were clustered together or not. For this, we used normalized DE gene counts from each sample. The PC1 and PC2 showed 44.1% (Fig. 2A) and 46.4% (Fig. 2C) variability between Sal-exposed and control groups in chicken heart and liver, respectively. The most dissimilarity between the groups was observed in the turkey heart (58.2%) (Fig. 2B). In contrast, the least variable groups were the turkey liver (40.6%) (Fig. 2D). As a result of PCA, more than 40% variability between the experimental and control groups was observed in both chicken and turkey heart and liver.

### Differential gene expression analysis

**Fig. 3:**
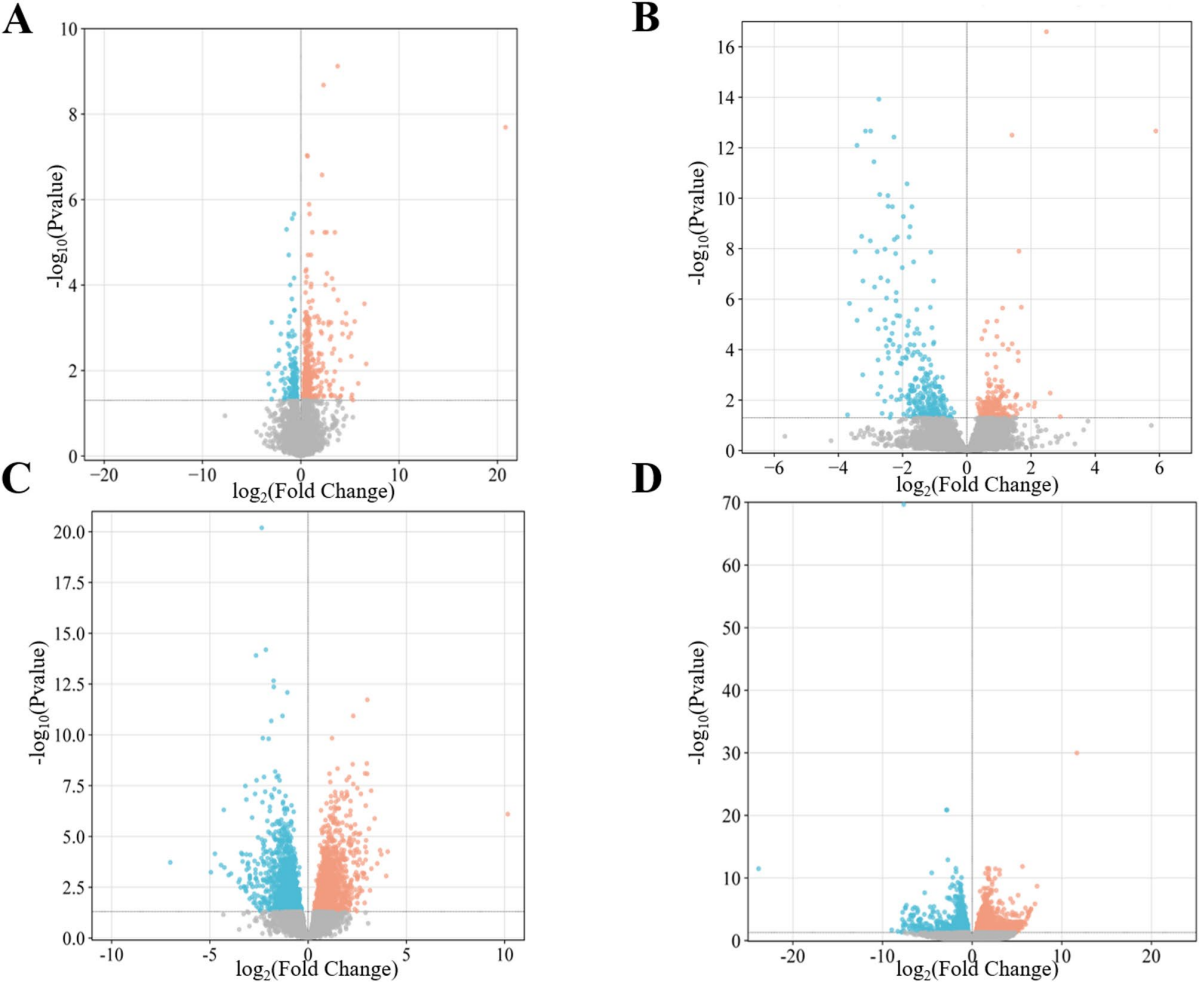
The volcano plot of DE genes for the heart and liver from chicken and turkey. Genes with Log_2_FC ≥ 1 and *p*-adj ≤ 0.05 were considered differentially expressed. Upregulated genes are shown in red, downregulated genes are shown in blue, and not significant genes (*p* adj > 0.05) are in grey (**A**: chicken heart, **B**: turkey heart, **C**: chicken liver, **D**: turkey liver.)

Differential gene expression analysis was performed to determine significantly changed genes between Sal-exposed and control groups. The cut-off values of DE genes were set as log_2_FC ≥ 1 and *p*-value ≤ 0.05 with FDR correction (*p*-adj). In the chicken heart, 673 DE genes were found between the Sal-exposed and control groups, of which 490 were upregulated and 183 were downregulated (Fig. 3A). In the case of chicken liver, 3049 DE genes were found between Sal-exposed and control groups, of which 1526 were upregulated and 1523 were downregulated (Fig. 3C). Similarly, 485 DE genes were determined between the groups in turkey heart, of which 188 DE genes were upregulated, whereas 297 DE genes were downregulated (Fig. 3B). In the case of turkey liver, 2337 DE genes were determined, of which 1169 were upregulated and 1168 were downregulated, respectively (Fig. 3D).

### GO analysis with chicken and turkey

**Fig. 4:**
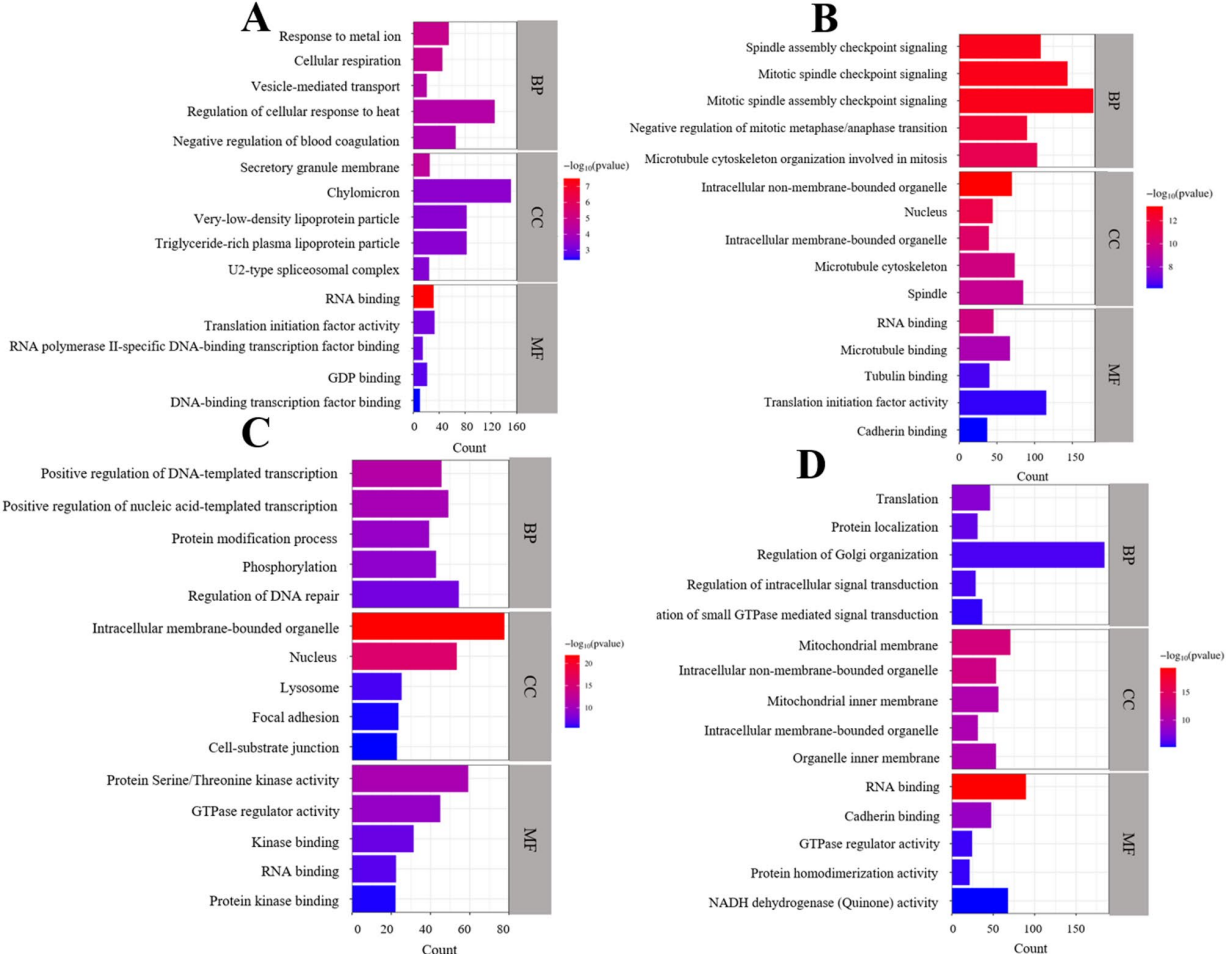
Gene Ontology (GO) analysis of DE genes in the heart and liver between the control and Sal-exposed groups for both species. GO terms were categorized into biological process (BP), cellular component (CC), and molecular function (MF), and ranked according to the *p*-value (*p* < 0.05). Bar plots show the top five enriched GO terms for each tissue and species: (**A**) chicken heart, (**B**) turkey heart, (**C**) chicken liver, and (**D**) turkey liver. Count represents the number of DE genes involved in each GO term.

We evaluated the top five GO terms (*p* < 0.05) in chicken and turkey. Terms were categorized as the biological process (BP), cellular component (CC), and molecular function (MF). Among the categories, the most significant terms were response to metal ion in BP, secretory membrane in CC, and RNA binding in MF for the chicken Sal-exposed heart group compared to the control group (Fig. 4A). In contrast, positive regulation of DNA-templated transcription (BP), intracellular membrane-bounded organelle (CC), and protein serine/threonine kinase activity (MF) were found as the most significant terms in chicken liver (Fig. 4C). The most significant terms in turkey heart were: spindle assembly checkpoint signaling (BP), intracellular non-membrane-bounded organelle (CC), and RNA binding (MF) (Fig. 4B). Translation (BP), mitochondrial membrane (CC), and RNA binding (MF) were the most significant enriched GO terms in turkey liver (Fig. 4D).

### Enrichment analysis of heart in chicken and turkey using Reactome

**Fig. 5:**
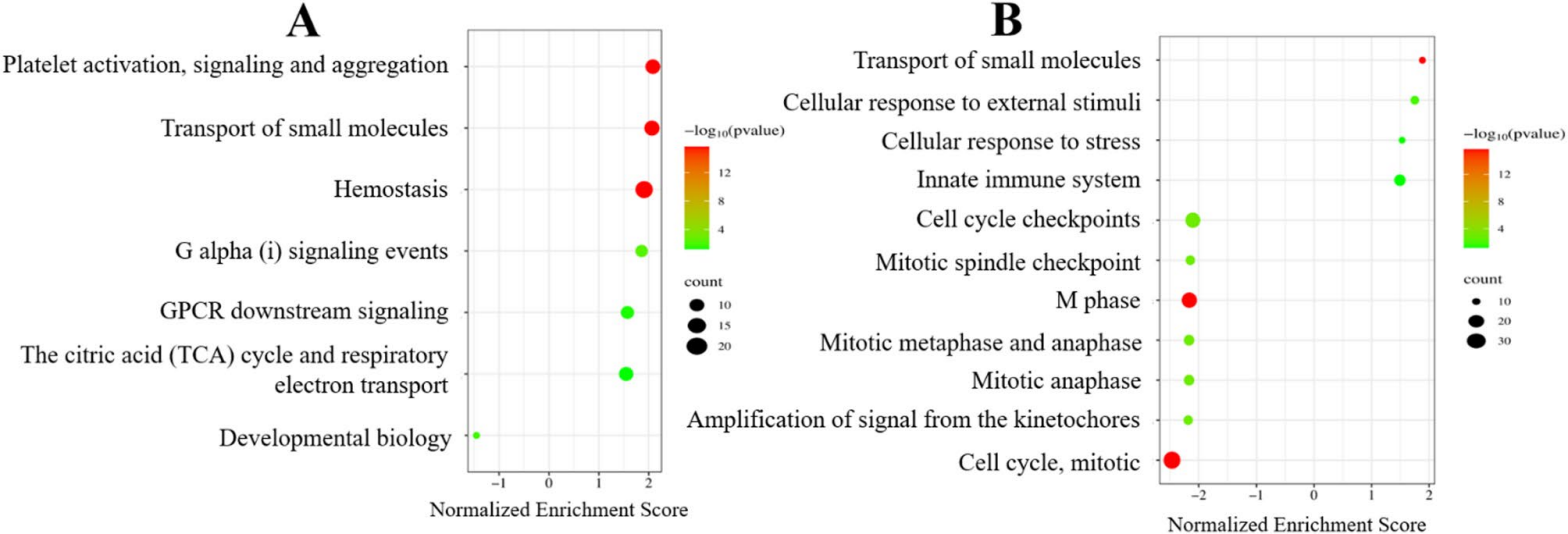
Reactome pathway enrichment analysis of DE genes in chicken heart (**A**) and turkey heart (**B**). Each bubble represents a significantly enriched pathway based on normalized enrichment score (NES). The X-axis shows the NES. Positive values indicate upregulation and negative values indicate downregulation in the Sal-exposed group compared to controls. Bubble size corresponds to the number of DE genes involved in each pathway (count), and bubble color indicates statistical significance as –log10(*p*-value).

The enrichment analysis has been performed with Reactome to identify molecular mechanisms of Sal toxicity using the putative biological roles of DE genes. Seven pathways were found to be significantly changed between Sal-exposed and control groups in the chicken heart. Six pathways were upregulated, whereas the developmental biology pathway showed downregulation. Platelet activation, signaling, and aggregation pathway (*p* < 2.2e−16), and transport of small molecules (*p* < 2.2e−16) were the most significant pathways. We determined increased regulation in hemostasis (*p* < 2.2e−16), G alpha signaling events (*p* = 0.004), and GPCR downstream (*p* = 0.03) signaling. Also, one of the energy metabolism pathways, the citric acid (TCA) cycle and respiratory electron transport (*p* = 0.05) pathway and involved genes were found to be upregulated (Fig. 5A). Reactome pathway analysis with DE genes in the turkey heart showed that 11 pathways (four upregulated and seven downregulated) were significantly altered between Sal-exposed and control groups. The most significant pathways have been determined as the transport of small molecules, M phase, and cell cycle (*p* < 2.2e−16). The innate immune system, cellular response to external stimuli, response to stress, and transport of small molecules were upregulated in the Sal-exposed group (Fig. 5B). The transport of small molecules has been found as a shared pathway between the chicken and turkey heart.

### Enrichment analysis of liver in chicken and turkey using Reactome

**Fig. 6:**
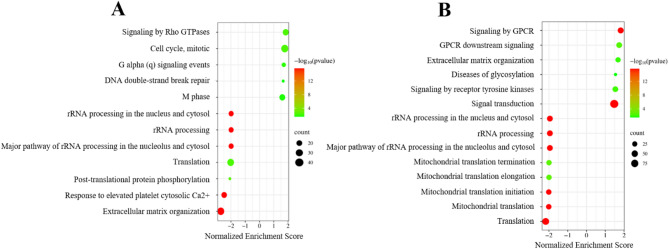
Reactome pathway enrichment analysis of DE genes in chicken liver (**A**) and turkey liver (**B**). Each bubble represents a significantly enriched pathway based on normalized enrichment score (NES). The X-axis shows the NES. Positive values indicate upregulation and negative values indicate downregulation in the Sal-exposed group compared to controls. Bubble size corresponds to the number of DE genes involved in each pathway (count), and bubble color indicates statistical significance as − log10(*p*-value).

We found that 12 pathways were significantly changed in the chicken liver Sal-exposed group: five were upregulated and seven were downregulated (Fig. 6A). The upregulated pathways were related to the cell cycle, cell division, and G signaling. The seven downregulated pathways were related to rRNA processing, translation, post-translation, extracellular matrix (ECM) organization, and platelet mechanism. In the turkey liver, 14 pathways were significantly enriched (six upregulated and eight downregulated) in the Sal-exposed group (Fig. 6B). The most enriched pathway was translation (*p* < 2.2e−16). According to the enrichment analysis results, the rRNA processing, translation, ECM organization, and G signaling were found to be shared pathways between chicken and turkey.

### Common DE genes between chicken and turkey

**Fig. 7:**
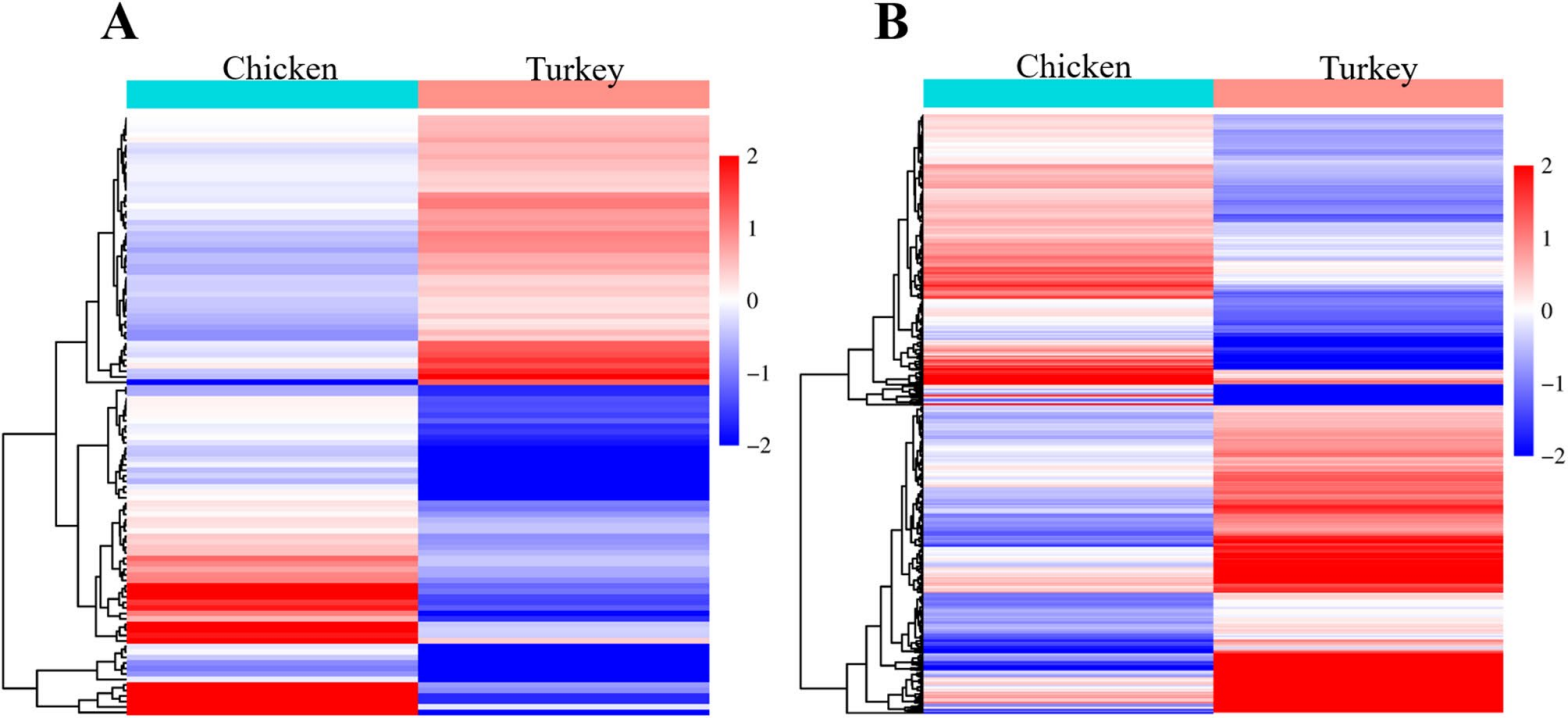
Heatmaps of common DE genes in chicken and turkey heart (**A**) and liver (**B**). Each heatmap represents Log_2_FC values for DE genes in both species. The vertical column represents tissues, and the horizontal column represents common DE genes with Log_2_FC values. Up and downregulated genes are illustrated as red and blue, respectively. Hierarchical clustering was performed using the complete linkage method with Euclidean distance.

We investigated common DE genes in the heart and liver between the Sal-exposed and control groups in chicken and turkey. All significant DE genes without cut-off restriction were included. We determined 109 common DE genes in the heart (Fig. 7A) and 795 common DE genes in the liver (Fig. 7B). The common DE genes showed distinct expression profiles between chicken and turkey. In the heart, severely downregulated common DE genes were observed in turkey, whereas their expressions were highly upregulated in chicken. In the liver, highly upregulated common DE genes were determined in turkey. Compared to the turkey liver, common DE gene expressions did not show severe alteration in the chicken liver. The expression profile of common DE genes in the heart and the liver is demonstrated with a heatmap.

### Validation of candidate DE genes in chicken with RT-qPCR

**Fig. 8:**
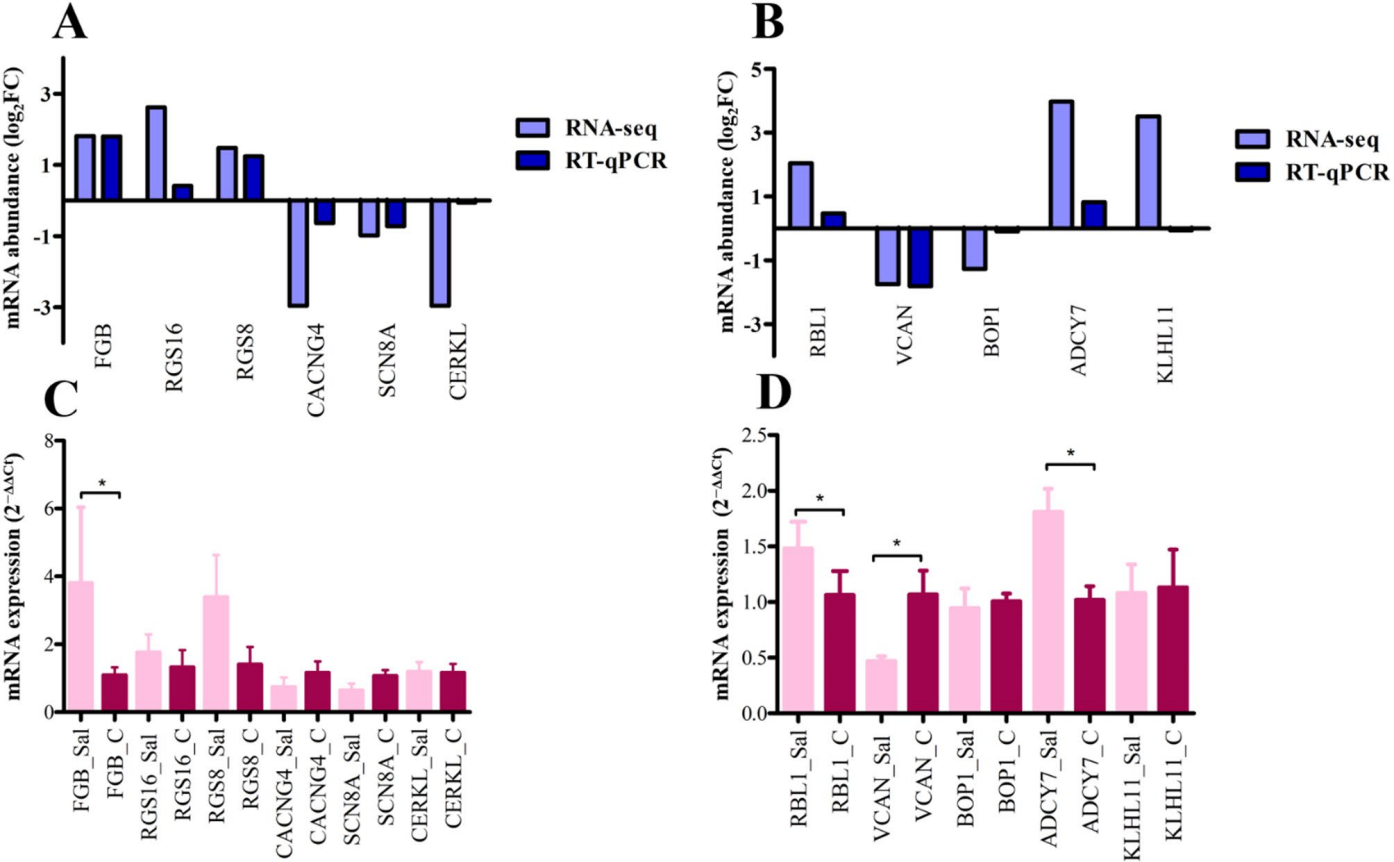
RNA-seq validation of chicken heart and liver with RT-qPCR. (**A**) Comparison of log_2_FC values between RNA-seq and RT-qPCR for six candidate genes in the chicken heart. (**B**) Comparison of log_2_FC values between RNA-seq and RT-qPCR for five candidate genes in the chicken liver. (**C**) RT-qPCR analysis of gene expression in the chicken heart between Sal-exposed and control groups using the 2^−ΔΔCt^ method. (**D**) RT-qPCR analysis of gene expression in the chicken liver between Sal-exposed and control groups using the 2^−ΔΔCt^ method. RT-qPCR data are presented as means ± SEM (n = 6), analyzed by unpaired *t-test*. ^*^p ≤ 0.05. Sal: Sal-exposed group; C: control group.

RNA-seq validation in the chicken heart was performed with the genes: fibrinogen beta chain (*FGB*), regulator of G protein signaling 16 (*RGS16*), regulator of G protein signaling 8 (*RGS8*), calcium voltage-gated channel auxiliary subunit gamma 4 (*CACNG4*), sodium voltage-gated channel alpha subunit 8 (*SCN8A*) and ceramide kinase like (*CERKL*). We found that in all candidate genes, the relative mRNA expression was correlated with RNA-seq (Fig. 8A). Between RNA-seq and RT-qPCR, the Spearman’s correlation coefficient and corresponding *p*-value detected as r = 0.66 *p* = 0.15 in the chicken heart.

Among the candidate genes for chicken liver RNA-seq validation, RB transcriptional corepressor like 1 (*RBL1*), versican (*VCAN*), BOP1 ribosomal biogenesis factor (*BOP1*), adenylate cyclase 7 (*ADCY7*) were found to be correlated with RNA-seq. In contrast, kelch like family member 11 (*KLHL11*) gene did not show a correlation (Fig. 8B). Between RNA-seq and RT-qPCR, the Spearman’s correlation coefficient and corresponding *p*-value detected as r = 0.9 *p* = 0.083 in chicken liver. The fold change and statistical significance of candidate genes were analyzed using RT-qPCR and calculated with the 2^−ΔΔCt^ method between the Sal-exposed and control groups. In chicken heart, only the *FGB* gene was significant. It was upregulated in the Sal-exposed group compared to the control group (Fig. 8C). In the chicken liver Sal-exposed group, *RBL1* and *ADCY7* genes were significantly upregulated, in contrast, *VCAN* was downregulated (Fig. 8D).

### Validation of candidate DE genes in turkey with RT-qPCR

**Fig. 9:**
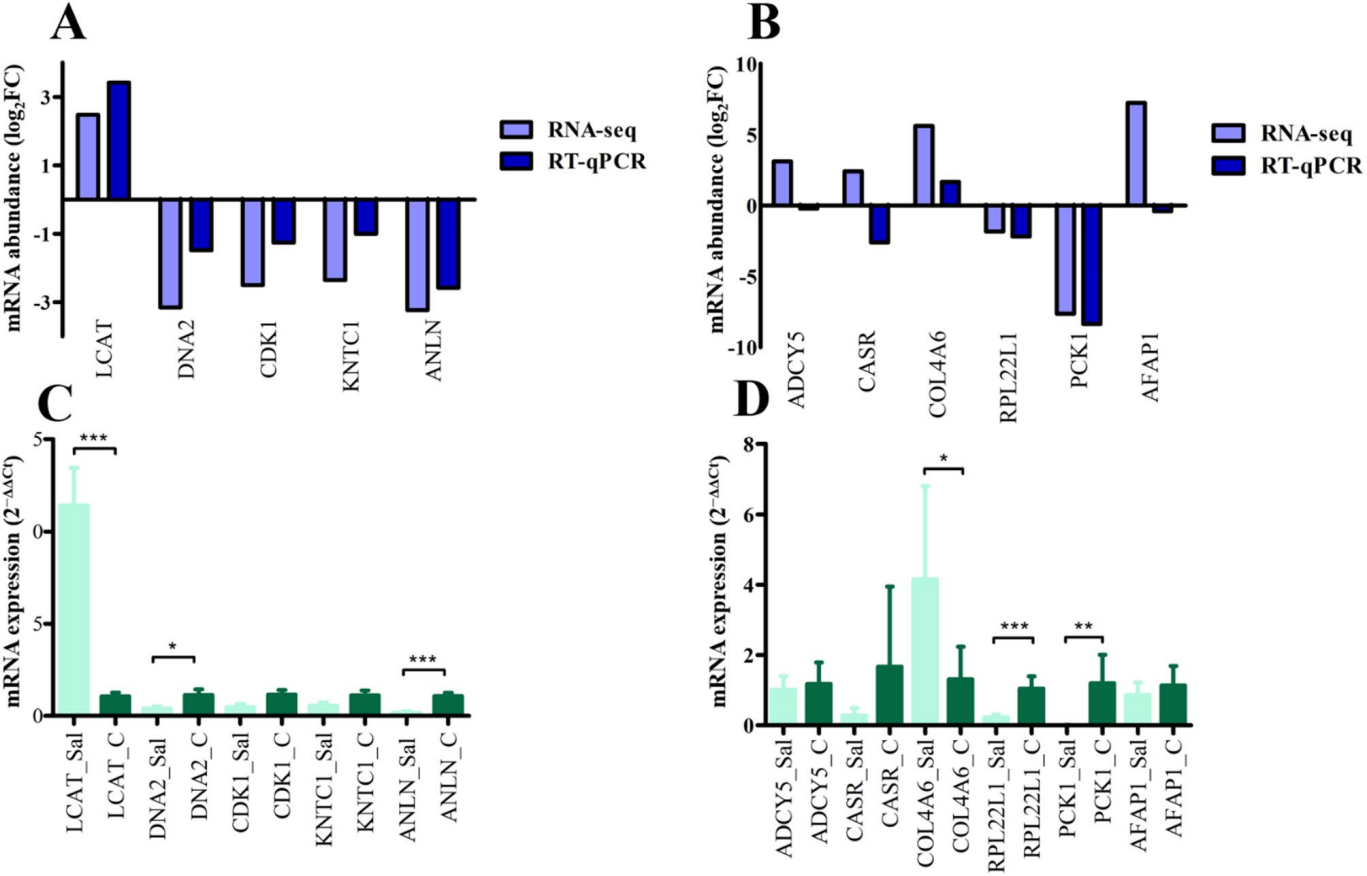
RNA-seq validation of turkey heart and liver with RT-qPCR. (**A**) Comparison of log_2_FC values between RNA-seq and RT-qPCR for five candidate genes in the turkey heart. (**B**) Comparison of log_2_FC values between RNA-seq and RT-qPCR for six candidate genes in the turkey liver. (**C**) RT-qPCR analysis of gene expression in the turkey heart between Sal-exposed and control groups using the 2^−ΔΔCt^ method. (**D**) RT-qPCR analysis of gene expression in the turkey liver between Sal-exposed and control groups using the 2^−ΔΔCt^ method. RT-qPCR data are presented as means ± SEM (n = 6), analyzed by unpaired *t-test*. ^*^p ≤ 0.05; ^**^p < 0.01; ***p < 0.001. Sal: Sal-exposed group; C: control group.

In turkey heart lecithin-cholesterol acyltransferase (*LCAT*), DNA replication helicase/nuclease 2 (*DNA2*), cyclin dependent kinase 1 (*CDK1*), kinetochore associated 1 (*KNTC1*) and anillin actin binding protein (*ANLN*) genes were selected for validation and for all those genes the RT-qPCR results correlated with RNA-seq (Fig. 9A). Between RNA-seq and RT-qPCR, the Spearman’s correlation coefficient and corresponding *p*-value detected as r = 0.97 *p* = 0.0048 in turkey heart.

In turkey liver among the six selected candidate genes collagen type IV alpha 6 chain (*COL4A6*), ribosomal protein L22 like 1 (*RPL22L1*) and phosphoenolpyruvate carboxykinase 1 (*PCK1*) showed a positive correlation, the adenylate cyclase 5 (*ADCY5*), calcium sensing receptor (*CASR*), and actin filament associated protein 1 (*AFAP1*) showed a negative correlation with RNA-seq data (Fig. 9B). Between RNA-seq and RT-qPCR, the Spearman’s correlation coefficient and corresponding *p*-value detected as r = 0.82 *p* = 0.046 in turkey liver. The fold change and statistical significance of candidate genes were analyzed using RT-qPCR and calculated with the 2^−ΔΔCt^ method between the Sal-exposed and control groups. Among five candidate genes in the turkey heart, the *LCAT* was significantly upregulated, whereas *DNA2* and *ANLN* were significantly downregulated in the Sal-exposed group (Fig. 9C). In turkey liver Sal-exposed group, *RPL22L1* and *PCK1* were significantly downregulated whereas *COL4A6* was upregulated (Fig. 9D).

## Discussion

The differences between chicken and turkey regarding Sal toxicity pose an issue for animal health, farmers, and consumers. Clinical findings on Sal toxicity are well established, but further investigation is needed to clarify its molecular mechanisms. In this study, we performed a series of experiments, including RNA-seq, gene set enrichment analysis, and RT-qPCR, to identify underlying molecular mechanisms of Sal toxicity in chicken and turkey heart and liver.

RNA-seq resulted in higher total and uniquely aligned reads in chicken compared to turkey. Chicken (INSDC Assembly: GCA_016699485.1), a well-studied model organism^47^, has almost 3.5 times higher genome coverage and 2.36 times more gene transcripts compared to turkey (INSDC Assembly: GCA_000146605.4). Therefore, the differences in genome assembly could explain why chickens have higher total and uniquely aligned reads. The number of significant DE genes in the liver was approximately 4.50 times higher than in the chicken heart and 4.81 times higher than in the turkey heart. Given that Sal is metabolized in the liver and impairs mitochondrial function^48–50^, the higher number of DE genes in the liver may be associated with metabolic processes. GO analysis in the heart pinpointed that GO terms were enriched with the cellular response, enzyme and RNA binding, and translation in chicken. In turkey, GO terms were related to cell cycle pathways, such as checkpoint signaling and microtubule regulation. Regarding liver tissue, the metabolic processes, such as transcription and enzyme activity, were determined in chicken, whereas GO terms were associated with energy metabolism in the turkey. The enriched GO terms differed for chicken and turkey. These results indicate species- and tissue-specific responses to Sal toxicity at the molecular level. We performed enrichment analysis with DE genes to determine the molecular pathways related to Sal toxicity between the control and Sal-exposed groups.

As a result of enrichment analysis in chicken heart, platelet activation, signaling and aggregation, transport of small molecules, G alpha signaling, TCA cycle, and respiratory electron transport were the most enriched pathways. Increased gene expression of ion channels supports the thesis that excessive Sal alters ion balance of the myocardial cells. Furthermore, it leads to increased intracellular Ca^2+^ levels in Sal-exposed group. As a key second messenger, increased Ca^2+^ may promote platelet activation—a known mediator to atherosclerosis and thrombosis, potentially leading to heart failure^51,52^. *FGB* gene upregulation was also observed, which has been associated with heart failure and attack^52,53^. Additionally, *CACNG4* and *SCN8A* were downregulated, which may impair myocardial contraction^54,55^. Downregulation of the TCA cycle and respiratory electron transport pathways suggests disrupted energy metabolism. Upregulation of *RGS16* and *RGS8*, regulators of G-protein–coupled signaling^56^, could promote the activation of G signaling in response to calcium-induced platelet activity.

To our knowledge, no comparative transcriptomic studies have investigated the molecular toxicity of Sal using an *in-vivo* approach. Gao et al.^13^ studied Sal cytotoxicity *in-vitro* and found apoptosis activation by triggered mitochondria dysfunction. Consistent with these findings, our *in-vivo* analysis showed that toxic Sal upregulated the apoptotic markers such as cytochrome c oxidase subunits I (*COX1*) and II (*COX2*) (Supplementary Table S4).

A study performed in 2018 investigated maduramicin (MAD) molecular toxicity in primary chicken myocardial cells. MAD is one of the dominantly used antiparasitic agents in chickens, and it shares a similar mode of action to Sal’s. It was demonstrated that toxic MAD altered cytokine-cytokine receptor interaction and calcium signal and induced mitochondria-associated apoptosis^25^. Supporting these findings, we found that, similar to MAD, toxic Sal also affected mitochondrial function, energy metabolism, and apoptosis in chicken hearts. We concluded that both Sal and MAD intoxication may dysregulate common molecular mechanisms in the heart. These pathways may be used as biomarkers for detecting Sal and MAD toxicity in chickens.

In the turkey heart, cell cycle–related pathways were downregulated, while immune and inflammatory pathways were upregulated in the Sal-exposed group. Studies demonstrated that Sal activates cell cycle arrest in various human cancers via downregulating mitotic checkpoint genes’ expression^50,57–59^. Similar to these cancer studies, we found that gene expressions of mitotic checkpoints, cyclins, and anti-apoptotic markers were downregulated in the Sal-exposed group (Supplementary Table S5). Apoptosis triggered by cell cycle arrest was observed only in the turkey heart, suggesting that cell cycle disruption may be a species-specific mechanism of Sal toxicity in turkeys. This response may also be associated with the upregulation of immune pathways. Heatmap analysis pinpointed distinct gene expression patterns between chicken and turkey hearts. Common downregulated DE genes correlated with the cell cycle, and their gene expression was severely decreased in turkey heart. In contrast, common upregulated DE genes played a role in platelet activation, and their gene expression was severely upregulated in chicken heart. These mechanisms indicate that Sal toxicity impairs different signaling pathways in the heart depending on the species.

In chicken liver, the enrichment analysis showed that translation, energy metabolism, and ECM organization were the most enriched pathways, and they were all downregulated in the Sal-exposed group. The study by Niwa et al.^60^ found that Sal-induced cell cycle arrest resulted in apoptosis via modulating the Cytochrome P450 (*CYP450*) family in HepG2/C3a cells. In addition to this study, the negative modulation of Sal was also demonstrated in rat liver^61^. Similar to these studies, we determined the downregulation of *CYP450* genes: *CYP3A4*, *CYP2C9*, *CYP2C23a*, *CYP2AC2*, *CYP2J2*, and *CYP4F11*. However, we found the upregulation of *CYP2U1* and *CYP7B1* in the Sal-exposed group. We concluded that Sal modulates its biotransformation in chicken liver like human and rat livers. The downregulation of *CYP3A4*, a major gene for Sal metabolism^60^, may show that the modulation occurs as inhibition and affects the cell’s energy metabolism, the mitochondria, in chicken liver.

As a result of enrichment analysis performed with turkey liver, the translation and energy metabolism pathways were downregulated in the Sal-exposed group. The decreased mRNA expression of *CYP21A1-2*, *CYP4B1*, *CYP8B1*, *CYP2C8*, *CYP4F8*, and *CYP4V2* showed that the Sal biotransformation mechanism was also impaired. Interestingly, there were no common CYP450 genes between chicken and turkey liver. We concluded that Sal toxicity may cause modulation of different gene expressions involved in the CYP450 family depending on the species. One of the upregulated pathways in turkey liver was ECM organization, which could be associated with promoting fibrosis. Liver fibrosis was also confirmed by histopathological analysis (Chłodowska, personal communication, June 2025). The heatmap analysis allowed us to evaluate expression patterns of common DE genes in chicken and turkey liver. Highly expressed common DE genes in the liver were found in the turkey and belonged to the ECM organization. In contrast, these genes showed either no change or lower expression in chickens. It indicates that even if the Sal may target the same DE genes across species, the magnitude of gene expression is species-dependent. On the other hand, the downregulated common DE genes were involved in mitochondrial pathways.

**Fig. 10:**
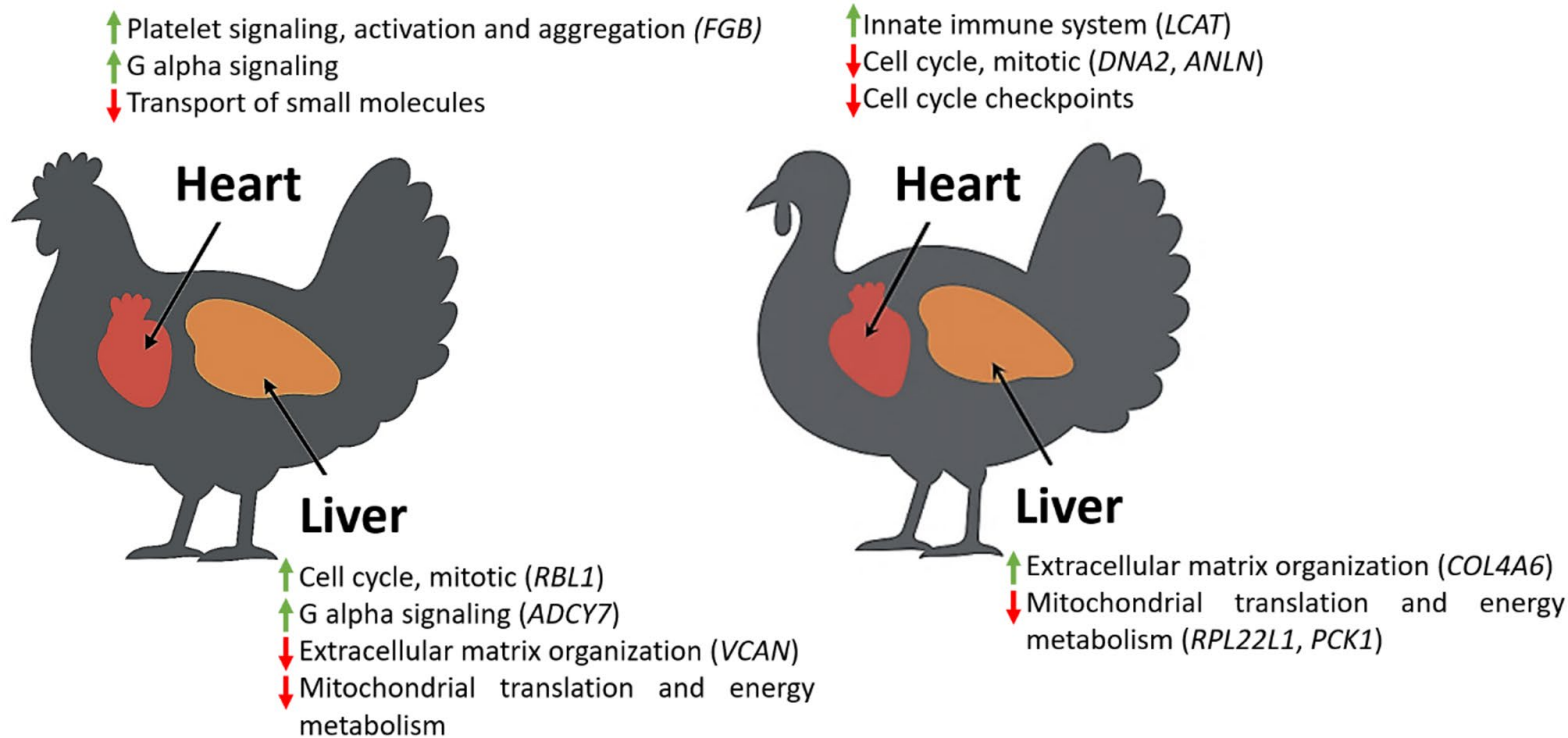
Conceptual diagram of Sal-induced molecular pathways in chicken and turkey heart and liver. This schematic illustrates the species- and tissue-specific molecular responses to Sal exposure, based on RNA-seq and pathway enrichment analyses. Pathways are annotated with representative DE genes in parentheses when those genes were significantly altered between the Sal-exposed and control groups based on RT-qPCR validation. (↑upregulated; ↓downregulated).

To provide a comprehensive overview of the species- and tissue-specific molecular responses to Sal toxicity, a conceptual diagram that summarizes the key enriched pathways and representative DE genes in chicken and turkey heart and liver was created (Fig. 10). All DE genes included in the model were significantly changed between the control and Sal-exposed groups and validated by RT-qPCR. This schematic illustrates how Sal exposure induces distinct transcriptional responses depending on species.

## Conclusion

This is the first comparative transcriptomics study using an *in-vivo* approach that explores Sal’s molecular toxicity mechanisms and investigates why turkeys are more susceptible to Sal toxicity than chickens. In conclusion, we show that Sal molecular toxicity affected distinct mechanisms depending on animal species and the tissues. Furthermore, we confirm that turkeys are more susceptible also at the molecular level.

## Electronic supplementary material

Below is the link to the electronic supplementary material.

Supplementary Material 1

Supplementary Material 2

Supplementary Material 3

Supplementary Material 4

Supplementary Material 5

## Data Availability

The sequence data have been deposited in the Gene Expression Omnibus (GEO) under accession numbers GSE289894 for chicken (https://www.ncbi.nlm.nih.gov/geo/query/acc.cgi?acc=GSE289894) and GSE289895 for turkey (https://www.ncbi.nlm.nih.gov/geo/query/acc.cgi?acc=GSE289895) data. The sequence and any other data are available from the corresponding author, Małgorzata Olejnik, upon request.
