# Supplementary material for: Comparative transcriptomics of salinomycin molecular toxicity in chicken and turkey
Source: Sci Rep. 2025 Jul 1;15:21586. doi: 10.1038/s41598-025-08812-7 (PMC12216427; doi:10.1038/s41598-025-08812-7)
Supplement: Supplementary file 1 — Supplementary Material 1 [file 41598_2025_8812_MOESM1_ESM.docx]

| Gene Symbol | Gene  Name | Accession Number | Forward  (5**^’^**-3’) | Reverse  (5**^’^**-3’) | Size  (bp) | Tm (^0^C) | Reference |
| --- | --- | --- | --- | --- | --- | --- | --- |
| FGB | Fibrinogen beta chain | NM_001167683.2 | CCAGATCCTTTTACCACGCCA | CTGAATCAAAGTCCAGCCTCCA | 75 | 58 | * |
| RGS16 | Regulator of G protein signaling 16 | NM_001277544.4 | GAGGATCCGATGAGTTGCTGG | TGAGTTCTTTGGCTCTCTCCA | 103 | 58 | 1 |
| RGS8 | Regulator of G protein signaling 8 | XM_046923296.1 | AACCGGGCTTTGAAAAGACTG | GTCTTCAGGAAAGCACGGAAAG | 113 | 56 | * |
| CACNG4 | Calcium voltage-gated channel auxiliary subunit gamma 4 | NM_204132.2 | TCAGAATACCTTCTCCGCATTGT | TAAATCCTCCCTGCTCCAACAC | 104 | 58 | * |
| SCN8A | Sodium voltage-gated channel alpha  subunit 8 | XM_040693922.2 | GTTTCAGCTCTGCGCACTTT | ACAAACTCTGTGACATATCTGGAAT | 79 | 60 | * |
| CERKL | Ceramide kinase like | XM_046921913.1 | TTCGAGATCGGCAAGACGAG | TGTACTGGAATCACCTGCGG | 99 | 58 | * |
| RBL1 | RB transcriptional corepressor like 1 | XM_040687910.2 | CTTCTGTGCTGCTTGGACCT | GGAAGTCAGCTGGTAAGCCTTT | 100 | 58 | * |
| VCAN | Versican | NM_204787.1 | GAGAATGCCGCCTACAAGGA | AGTCGTGCCCAATACGGTTC | 84 | 58 | * |
| BOP1 | BOP1 ribosomal biogenesis factor | XM_040665282.2 | CAGAGGAAGATGAGGGTGAACG | TCGGAGCCTGATGCCAAC | 173 | 58 | 2 |
| ADCY7 | Adenylate cyclase 7 | XM_040681125.2 | TCGACTTCACAAGGAAGGCA | GCCACACCATGGGTTAGTTG | 165 | 58 | * |
| KLHL11 | Kelch like family member 11 | XM_040691837.2 | TGGAGATGGCCGACAGGTTC | CTGTGGAAGTTCCTGCGGAT | 172 | 58 | * |
| LCAT | Lecithin-cholesterol acyltransferase | XM_010717864.3 | TCTGGCTCAACCTCAACACG | GTTCGGTTGTACACTACCCTGG | 79 | 58 | * |
| DNA2 | DNA replication helicase/nuclease 2 | XM_019617937.2 | GAAGAGTTCATGCACAATCCAGC | CTGTTTTGTCTTCACTTGGCAGTT | 70 | 58 | * |
| CDK1 | Cyclin dependent kinase 1 | XM_003207855.4 | CAGGCCAAGTGGTTGCAATG | TGCATAAGAACATCCTGAAGACAGA | 139 | 58 | * |
| KNTC1 | Kinetochore associated 1 | XM_019620916.2 | TGGCTTAGGCATCAGGCAG | TGGCATTAAGCAAGCTCTCCA | 70 | 58 | * |
| ANLN | Anillin actin binding protein | XM_010713033.3 | CACCCTTCTTACTCCAGCCAT | TCCCCTTCAAAATTCATCTTGTCCA | 147 | 58 | * |
| ADCY5 | Adenylate cyclase 5 | XM_019617404.2 | CCAAGCAGGTGGATGACAGA | CAGCATAAATGTAGAGTGCGGC | 110 | 58 | * |
| CASR | Calcium sensing receptor | XM_031555809.1 | GATTCTCAAAGGAGGTGCCCTT | GGCAGTCCACACACTCGAAG | 111 | 58 | * |
| COL4A6 | Collagen type IV alpha 6 chain | XM_031554736.1 | CAGGACCAAAAGGGCTGGAT | TCCCTTGATGCCCAAAGGAC | 131 | 58 | * |
| RPL22L1 | Ribosomal protein L22 like 1 | XM_010717084.2 | TGATTCCGGGAACTTTGAACAG | AGTGTTCCCCAAGTTTCCAGTT | 76 | 58 | * |
| PCK1 | Phosphoenolpyruvate carboxykinase 1 | XM_003212123.4 | ATTGACGAGCCATTACCCCC | ATACAAGAGGCACACCAAGCAG | 217 | 60 | * |
| AFAP1 | Actin filament associated protein 1 | XM_031553213.1 | CATCAAGCCCACAGTCTCCA | TGTTTGATGAGGGAGGTCGC | 138 | 58 | * |
| G6PDH | Glucose-6-phosphate dehydrogenase | AI981686.1 | CGGGAACCAAATGCACTTCGT | GGCTGCCGTAGAGGTATGGGA | 122 | 58 | 3 |
| ACTB | Actin beta | NM_205518.2 | CACAGATCATGTTTGAGACCTT | CATCACAATACCAGTGGTACG | 101 | 58 | 4 |
| PGK1 | Phosphoglycerate kinase | 100550807 | CAAAGGCCCTTGAGAGTCCA | ATGCCATTCCACCACCAATG | 132 | 58 | 5 |
| SDHA | Succinate dehydrogenase complex flavoprotein  subunit A | 100546340 | CTAAGCCACTCCAAGGCCAA | ATAGGAGCGAATAGCAGGCG | 173 | 58 | 5 |

**Supplementary Table S1.** Details of selected target and housekeeping genes in heart and liver.

^*^Designed in this study with Primer-BLAST (https://www.ncbi.nlm.nih.gov/tools/primer-blast/).

**Supplementary Table S1.** Twenty-two DE genes were selected to validate the reliability of RNA-seq data using RT-qPCR. The RefFinder tool was used to choose housekeeping genes. FGB, RGS16, RGS8, CACNG4, SCN8A, and CERKL genes were selected for chicken heart, whereas RBL1, VCAN, BOP1, ADCY7 and KLHL11 genes were selected for chicken liver. LCAT, DNA2, CDK1, KNTC1 and ANLN genes were selected for turkey heart, whereas ADCY5, CASR, COL4A6, RPL22L1, PCK1 and AFAP1 genes were selected for turkey liver. G6PDH and ACTB housekeeping genes were used for chicken heart and liver. PGK1 and SDHA housekeeping genes were used for the turkey heart and liver.

References

1. Ye, M., *et al*. Exploring the association between fat-related traits in chickens and the RGS16 gene: insights from polymorphism and functional validation analysis. *Front. Vet. Sci.* **10**, 1180797 (2023).
2. Zhu, H., *et al*. RNA-seq identifies differentially expressed genes involved in csal1 overexpression in granulosa cells of prehierarchical follicles in Chinese Dagu hens. *Poult. Sci.* **102**, 102310 (2023).
3. Sevane, N., *et al*. Dietary inulin supplementation modifies significantly the liver transcriptomic profile of broiler chickens. *PLoS ONE*. 9, e98942 (2014).
4. De Boever, S., Vangestel, C., De Backer, P., Croubels, S. & Sys, S.U. Identification and validation of housekeeping genes as internal control for gene expression in an intravenous LPS inflammation model in chickens. *Vet. Immunol. Immunopathol.* **122**, 312–317 (2008).
5. Brady, K., Liu, H. C., Hicks, J., Long, J. A., & Porter, T. E. Global gene expression analysis of the turkey hen hypothalamo-pituitary-gonadal axis during the preovulatory hormonal surge. *Poult. Sci.* **102**, 102547 (2023).
