# Supplementary material for: Comparative transcriptomics of salinomycin molecular toxicity in chicken and turkey
Source: Sci Rep. 2025 Jul 1;15:21586. doi: 10.1038/s41598-025-08812-7 (PMC12216427; doi:10.1038/s41598-025-08812-7)
Supplement: Supplementary file 2 — Supplementary Material 2 [file 41598_2025_8812_MOESM2_ESM.docx]

**Supplementary Table S2.** Quality control report of RNA-seq in chicken heart and liver.

| **Sample Name** | **Sample Code** | **Tissue** | **Total Reads**  **(M)** | **Uniquely Aligned (M)** | **% > Q30** | **GC (%)** |
| --- | --- | --- | --- | --- | --- | --- |
| 01T0001 | CHR_SAL_exposed_1 | Heart | 44310632 | 40754316 (94.2%) | 94.1% | 48.0% |
| 01T0002 | CHR_SAL_exposed_2 | Heart | 43210472 | 39855292 (93.9%) | 94.4% | 49.2% |
| 01T0003 | CHR_SAL_exposed_3 | Heart | 53503266 | 48162624 (91.2%) | 94.9% | 49.8% |
| 01T0004 | CHR_SAL_exposed_4 | Heart | 44933439 | 41407499 (94.5%) | 93.8% | 47.5% |
| 01T0005 | CHR_SAL_exposed_5 | Heart | 48242442 | 44258287 (93.0%) | 94.6% | 49.0% |
| 01T0006 | CHR_SAL_exposed_6 | Heart | 61169422 | 56683847 (94.0%) | 94.7% | 49.0% |
| 01T0019 | CHR_Control_1 | Heart | 48869082 | 44065271 (91.4%) | 94.1% | 49.1% |
| 01T0020 | CHR_Control_2 | Heart | 58160678 | 53566302 (93.6%) | 94.1% | 48.9% |
| 01T0021 | CHR_Control_3 | Heart | 50012282 | 45888006 (93.0%) | 94.1% | 49.0% |
| 01T0022 | CHR_Control_4 | Heart | 46876032 | 42275256 (91.3%) | 94.3% | 49.1% |
| 01T0023 | CHR_Control_5 | Heart | 58807540 | 53622974 (92.4%) | 94.2% | 49.1% |
| 01T0024 | CHR_Control_6 | Heart | 56048337 | 50875174 (92.0%) | 94.1% | 49.4% |
| 01T0013 | CLV_SAL_exposed_1 | Liver | 52289869 | 44241125 (85.8%) | 95.2% | 51.3% |
| 01T0014 | CLV_SAL_exposed_2 | Liver | 52871142 | 44834842 (86.1%) | 95.1% | 52.3% |
| 01T0015 | CLV_SAL_exposed_3 | Liver | 54389725 | 47465671 (88.4%) | 95.1% | 50.7% |
| 01T0016 | CLV_SAL_exposed_4 | Liver | 48208014 | 41897965 (90.0%) | 94.4% | 48.2% |
| 01T0017 | CLV_SAL_exposed_5 | Liver | 46511650 | 40465270 (88.2%) | 94.5% | 50.2% |
| 01T0018 | CLV_SAL_exposed_6 | Liver | 46215630 | 40133853 (88.0%) | 94.3% | 50.0% |
| 01T0031 | CLV_Control_1 | Liver | 49319861 | 41667372 (88.4%) | 94.6% | 50.2% |
| 01T0032 | CLV_Control_2 | Liver | 49329184 | 40614899 (83.9%) | 94.2% | 52.8% |
| 01T0033 | CLV_Control_3 | Liver | 48293048 | 40504883 (85.6%) | 93.9% | 51.7% |
| 01T0034 | CLV_Control_4 | Liver | 44556216 | 37245811 (85.9%) | 93.9% | 50.3% |
| 01T0035 | CLV_Control_5 | Liver | 51835893 | 42302248 (85.1%) | 94.3% | 50.7% |
| 01T0036 | CLV_Control_6 | Liver | 57898257 | 46267038 (85.3%) | 94.2% | 48.4% |

M: Millions.

**Supplementary Table S2.** The initial sequence data underwent quality assessment using FastQC. The cleaned sequences were then mapped to the Gallus gallus genome (bGalGal1.mat.broiler.GRCg7b) using reference annotations that included 17,007 genes from the Ensembl database for chicken. Alignment was produced with RNA-SeQC. The MultiQC tool was used to visualize raw sequence data and compile results into a single report. Uniquely aligned reads varied between 84% and 94%. For each sample, the % >Q30 was above 93%. The sequence data have been deposited in the Gene Expression Omnibus (GEO) under accession numbers GSE289894.
