# Supplementary material for: Comparative transcriptomics of salinomycin molecular toxicity in chicken and turkey
Source: Sci Rep. 2025 Jul 1;15:21586. doi: 10.1038/s41598-025-08812-7 (PMC12216427; doi:10.1038/s41598-025-08812-7)
Supplement: Supplementary file 3 — Supplementary Material 3 [file 41598_2025_8812_MOESM3_ESM.docx]

**Supplementary Table S3.** Quality control report of RNA-seq in turkey heart and liver.

| **Sample Name** | **Sample Code** | **Tissue** | **Total Reads**  **(M)** | **Uniquely Aligned**  **(M)** | **% > Q30** | **GC (%)** |
| --- | --- | --- | --- | --- | --- | --- |
| 01T0037 | THR_SAL_exposed_1 | Heart | 42000876 | 33802834 (81.6%) | 95.2% | 49.3% |
| 01T0038 | THR_SAL_exposed_2 | Heart | 46291919 | 36800014 (80.2%) | 94.9% | 47.7% |
| 01T0039 | THR_SAL_exposed_3 | Heart | 51361806 | 41124133 (81.7%) | 94.7% | 44.8% |
| 01T0040 | THR_SAL_exposed_4 | Heart | 47711170 | 37347806 (80.4%) | 93.5% | 44.7% |
| 01T0041 | THR_SAL_exposed_5 | Heart | 50090480 | 40164697 (81.2%) | 95.2% | 48.3% |
| 01T0042 | THR_SAL_exposed_6 | Heart | 52909173 | 41918596 (80.1%) | 94.8% | 47.9% |
| 01T0055 | THR_Control_1 | Heart | 53436223 | 42458730 (80.4%) | 94.9% | 47.6% |
| 01T0056 | THR_Control_2 | Heart | 44213189 | 34243254 (79.0%) | 94.3% | 44.7% |
| 01T0057 | THR_Control_3 | Heart | 45451076 | 36137728 (80.7%) | 94.9% | 47.9% |
| 01T0058 | THR_Control_4 | Heart | 49795449 | 39440184 (80.4%) | 93.7% | 47.7% |
| 01T0059 | THR_Control_5 | Heart | 48089098 | 37777231 (79.3%) | 94.4% | 47.6% |
| 01T0060 | THR_Control_6 | Heart | 47620660 | 35976208 (79.2%) | 94.4% | 47.3% |
| 01T0049 | TLV_SAL_exposed_1 | Liver | 49030518 | 38420992 (80.5%) | 94.5% | 46.5% |
| 01T0050 | TLV_SAL_exposed_2 | Liver | 44026979 | 36392884 (84.2%) | 94.9% | 47.5% |
| 01T0051 | TLV_SAL_exposed_3 | Liver | 42240191 | 34426661 (83.3%) | 94.5% | 48.8% |
| 01T0052 | TLV_SAL_exposed_4 | Liver | 43875135 | 36058199 (84.0%) | 95.2% | 48.6% |
| 01T0053 | TLV_SAL_exposed_5 | Liver | 50269686 | 41602736 (83.9%) | 95.1% | 48.9% |
| 01T0054 | TLV_SAL_exposed_6 | Liver | 52993535 | 43537179 (84.9%) | 94.4% | 45.8% |
| 01T0067 | TLV_Control_1 | Liver | 54391307 | 40829429 (78.7%) | 94.3% | 47.6% |
| 01T0068 | TLV_Control_2 | Liver | 51738714 | 38051046 (76.2%) | 94.5% | 47.9% |
| 01T0069 | TLV_Control_3 | Liver | 9478744 | 6153715 (76.7%) | 93.5% | 45.1% |
| 01T0070 | TLV_Control_4 | Liver | 56848305 | 41328918 (79.5%) | 94.9% | 46.4% |
| 01T0071 | TLV_Control_5 | Liver | 47214172 | 35581868 (78.3%) | 93.9% | 48.6% |
| 01T0072 | TLV_Control_6 | Liver | 50793497 | 34533591 (69.6%) | 94.2% | 52.0% |

M: Millions. The 01T0069 sample has been excluded from this study.

**Supplementary Table S2.** The initial sequence data underwent quality assessment using FastQC. The cleaned sequences were then mapped to the Meleagris gallopavo genome (Turkey_5.1.110) using reference annotations that included 16,226 genes from the Ensembl database for turkey. Alignment was produced with RNA-SeQC. The MultiQC tool was used to visualize raw sequence data and compile results into a single report. Uniquely aligned reads varied between 70% and 82%. For each sample, the % >Q30 was above 94%. The 01T0069 sample from the liver control group did not provide high-quality data (9.4 million total reads and 6.1 million unique alignments). This sample was excluded from the study. The sequence data have been deposited in the Gene Expression Omnibus (GEO) under accession numbers GSE289895.
